# Dynamic Changes of Cytoskeleton-Related Proteins Within Reward-Related Brain Regions in Morphine-Associated Memory

**DOI:** 10.3389/fnins.2020.626348

**Published:** 2021-01-28

**Authors:** Xixi Yang, Yichong Wen, Yuxiang Zhang, Feifei Gao, Jingsi Yang, Zhuojin Yang, Chunxia Yan

**Affiliations:** ^1^College of Forensic Medicine, Xi’an Jiaotong University Health Science Center, Xi’an, China; ^2^Bio-Evidence Sciences Academy, Western China Science and Technology Innovation Harbor, Xi’an Jiaotong University, Xi’an, China; ^3^NHC Key Laboratory of Drug Addiction Medicine, Kunming Medical University, Kunming, China

**Keywords:** morphine, extinction, β-actin, Arc/Arg31, ERK, NAc, dorsal hippocampus

## Abstract

Drug-induced memory engages complex and dynamic processes and is coordinated at multiple reward-related brain regions. The spatiotemporal molecular mechanisms underlying different addiction phases remain unknown. We investigated the role of β-actin, as well as its potential modulatory protein activity-regulated cytoskeletal-associated protein (Arc/Arg3.1) and extracellular signal-regulated kinase (ERK), in reward-related associative learning and memory using morphine-induced conditioned place preference (CPP) in mice. CPP was established by alternate morphine (10 mg/kg) injections and extinguished after a 10-day extinction training, while the withdrawal group failed to extinguish without training. In the nucleus accumbens (NAc), morphine enhanced the level of β-actin and Arc only during extinction, while p-ERK1/2 was increased during both CPP acquisition and extinction phases. In the dorsal hippocampus, morphine induced an upregulation of p-ERK only during extinction, while p-β-actin was elevated during both CPP establishment and extinction. In the dorsal hippocampus, Arc was elevated during CPP formation and suppressed during extinction. Compared with the NAc and dorsal hippocampus, dynamic changes in the medial prefrontal cortex (mPFC) and caudate putamen (CPu) were not very significant. These results suggested region-specific changes of p-β-actin, Arc/Arg3.1, and p-ERK1/2 protein during establishment and extinction phases of morphine-induced CPP. These findings unveiled a spatiotemporal molecular regulation in opiate-induced plasticity.

## Introduction

Drug addiction is a brain disease, defined as compulsive drug use, and characterized by stubborn persistence despite adverse consequences (Hyman, 2005; Zhang et al., 2018). Long-term alterations in brain neural circuits are well known to be responsible for normal appetitive learning and memory processes (Torregrossa et al., 2011). Drug-associated memory is an aberrant memory that shares the same memory processes with other forms of memories (Liu et al., 2019). The medial prefrontal cortex (mPFC) and hippocampus are involved in memory encoding and storage. The striatum is a subcortical structure in the forebrain, which can be further subdivided into dorsal [caudate putamen (CPu)] and ventral [nucleus accumbens (NAc)], and is implicated in regulating responses to rewarding stimuli (Wang et al., 2019; Bang et al., 2020; Spechler et al., 2020). Exposure to addictive drugs has been shown to induce both structural and functional changes in these brain regions (Kai et al., 2018).

Drug addiction is associated with large mushroom-shaped changes in synaptic plasticity of spines (Rothenfluh and Cowan, 2013). In this regard, cytoskeletal actin is the major structural component of the dendritic spine and has been shown to play a role in synaptic plasticity by maintaining characteristically and highly dynamic transformation through actin rearrangement (Hou et al., 2009). It is thus meaningful that the drug abuse field has focused on drug-induced changes in cytoskeletal protein and its regulatory mechanisms. Recent studies found that the actin cytoskeleton has been shown to be involved in many key neuronal processes by subserving events related to memory at different stages of learning and memory (Lamprecht, 2016; Basu and Lamprecht, 2018; Henneberger et al., 2020; Suratkal et al., 2020). One of the cytoskeletal proteins, β-actin, has already been found to change drastically after morphine administration. Activity-regulated cytoskeletal-associated protein (Arc), or Arg3.1, is an important cytoskeletal protein (Bramham et al., 2010), which is able to maintain F-actin stability and contributes to its polarized elongation by binding to it. Activating the brain-derived neurotrophic factor (BDNF) TrkB receptor, group 1 metabotropic glutamate receptors (mGluR1s), and *N*-methyl-D-aspartate (NMDA) receptors dramatically upregulated Arc transcription. The extracellular signal-regulated kinase (ERK) is a central node of the signaling pathways downstream of these receptors and is required for Arc transcription (Korb and Finkbeiner, 2011). However, their intimate relationship in morphine-induced memory remained to be elucidated.

In the present study, using conditioned place preference (CPP) to measure drug-associated reward memory, we focused on the regulation of drug reward memories by modulating two phases of memory: acquisition and extinction. Besides, we also compared extinction training and withdrawal. Lastly, we explored the effects of actin-related protein expression in different phases of morphine-induced CPP in relevant brain regions.

## Materials and Methods

### Animals and Drugs

Adult male C57BL/6J mice weighing 25–30 g were purchased from Xi’an Jiaotong University Animal Laboratory. Ninety-six mice were housed under constant temperature (22–24°C) and humidity (50–60%) and maintained on a 12-h light/dark cycle (lights on at 8 a.m.). Food and water were available *ad libitum*. All mice were handled individually with a sham intraperitoneal (i.p.) injection once daily for a week. The experimental procedures were approved by Xi’an Jiaotong University Laboratory Animal Administration Committee and conducted in accordance with the Xi’an Jiaotong University Guidelines for Animal Experimentation. Morphine hydrochloride was purchased from First Pharmaceutical Factory of Shenyang (China), dissolved in sterile 0.9% saline, and administered by i.p. injection at a volume of 10 mg/kg.

### Behavioral Test

**FIGURE 1:**
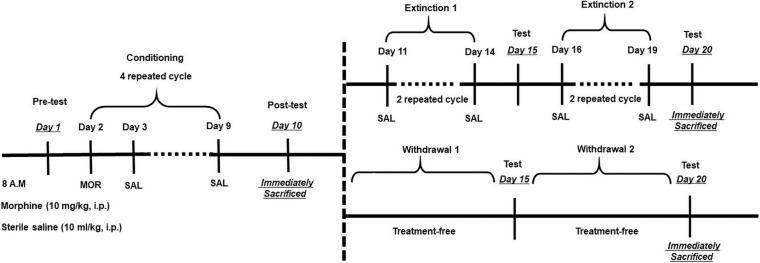
Experimental design for morphine conditioned place preference (CPP) and extinction training. After habituation for 1 week, pretest was performed on Day 1 for an unconditioned place preference. Conditioning period consists of four repeated cycles (Days 2–9), followed by a posttest on Day 10. Then, mice were treated with two kinds of extinction. As for the training extinction group, mice were trained through two trials, Trial 1 (Days 11–14) and Trial 2 (Days 16–19); during extinction trials, mice were placed in one chamber and, on alternate days, the other chamber with both saline [intraperitoneal (i.p.)]. As for the withdrawal group, mice were put in their homecage without any treatment. Tests for CPP expression were performed after each extinction trial on Days 15 and 20. On posttest Day 10 and extinction Day 20, mice were sacrificed by decapitation immediately after the test for protein analysis.

The CPP procedures were similar with previous study with some modifications, and detailed procedures were divided into two phases as shown in Figure 1. We focused on two phases of memory, i.e., acquisition and extinction.

#### Acquisition of Morphine Conditioned Place Preference

After 1 week of habituation, a total of 96 mice randomly divided into two equal groups (*n* = 48) were used for CPP. On day 1, all mice were placed separately into the apparatus for 15 min with free access to all the compartments, and the amount of time spent in each compartment was measured to assess unconditioned preference. The mice that spent more than 60% (>540 s) of the total time (900 s) for either of the compartments were eliminated from the study. During the conditioning phase (days 2–9), mice in the morphine treatment group were treated once daily from 8:00 a.m. for 8 consecutive days with four cycles of morphine (10 mg/kg, i.p.), then saline (10 ml/kg, i.p.) on alternating days. After the injection of morphine or saline, mice were immediately placed in the white compartment or black compartment for 40 min with sliding door. In the control group, saline was given in all training days with alternating compartment. On the 10th day, the sliding door was removed, and mice were allowed to move freely for 15 min. CPP score was determined by the time spent in morphine-paired compartment minus the time in saline-paired compartment. After the posttest, six mice were eliminated from the morphine group for the lack of formation CPP, while four mice were eliminated from the saline control group for the sharp variation from the baseline test. In addition, data about the moving distance, average velocity, and shuttle times were also collected.

#### Extinction of Morphine Conditioned Place Preference

In the extinction phase, mice in the morphine group (*n* = 42) were randomly divided into two groups and respectively underwent two extinction sessions, i.e., *Extinction 1* (days 11–14) and *Extinction 2* (days 16–19). Each session was followed by a test. For training extinction (Training Ext) groups (*n* = 21), mice were confined to each chamber for 40 min on alternate days with i.p. saline. For withdrawal groups (*n* = 21), mice were put in their homecage without any treatment. The remaining mice in the saline group (*n* = 44) were randomly divided into two groups (*n* = 22) as control of the above two experiment groups separately. Tests for CPP expression were performed after each extinction trial on days 15 and 20 for both groups. The success judgment of extinction training is based on the fact that CPP scores in 2 consecutive days during the extinction period became equal to those on the preconditioning test.

### Experimental Procedures for Western Blotting

Animals were divided into three main groups as morphine-induced CPP group (*n* = 16), training extinction group (*n* = 16), and withdrawal group (*n* = 16). Each group consists of CPP acquisition (*n* = 8) and saline control (*n* = 8). For saline and morphine-induced CPP groups, animals were sacrificed on day 10, and the training extinction and withdrawal groups were on day 20. Immediately after the test, the NAc, dorsal hippocampus, CPu, and mPFC were dissected out for Western blotting. Tissues were homogenized in an ice-cold radioimmunoprecipitation assay (RIPA) buffer (Pioneer) containing protease phosphatase inhibitor cocktail (Roche). Samples were centrifuged for 20 min at 12,000 rpm at 4°C. Protein concentrations were determined using the bicinchoninic acid (BCA) assay (Pioneer) and analyzed directly by sodium dodecyl sulfate-polyacrylamide gel electrophoresis (SDS-PAGE). Membrane homogenates (10 μg) were loaded in 12% SDS-PAGE gels, transferred onto polyvinylidene fluoride (PVDF) membranes. Membranes were blocked in TBST [Tris-buffered saline (TBS) containing 0.1% Tween 20] containing 5% non-fat dry milk for 2 h. Then, blots were incubated overnight at 4°C with specific antibodies against p-ERK1/2 (1:1,000; Cell Signaling Technology), total ERK1/2 (1:1,000; Cell Signaling Technology), p-Actin (p-Actin, 1:1,000; EMC Biosciences), total β-actin (1:1,000; Santa Cruz), and Arc/Arg3.1 (1:1,000; Proteintech). Then, the PVDF membrane was incubated with goat anti-rabbit or anti-mouse horseradish peroxidase-conjugated secondary antisera (1:10,000) for 120 min. Blots were developed with an enhanced chemiluminescence (ECL) plus detection kit (Millipore Corporation, Bedford, MA, United States). β-Actin was used as a loading control. The blot intensities were analyzed using ImageJ (NIH) to calculate the target protein.

### Data Analysis

All behavioral and molecular data were expressed as the mean ± SEM. The data from CPP test were analyzed with Student’s *t*-test, two-way ANOVA; the different protein expression levels were analyzed with one-way ANOVA followed by Bonferroni *post hoc* analysis for multiple comparisons. For results with significant interaction effects in two-way ANOVA, a simple effect test was performed for further analysis. For results without significant interaction effects, Bonferroni *post hoc* test or Student’s *t*-test was further conducted as needed. The data analyses were performed using IBM SPSS statistics 24 and GraphPad Prism 7.0. Statistical significance was set at *p* < 0.05.

## Results

### Behavioral Experiment

#### Acquisition of Conditioned Place Preference

**FIGURE 2:**
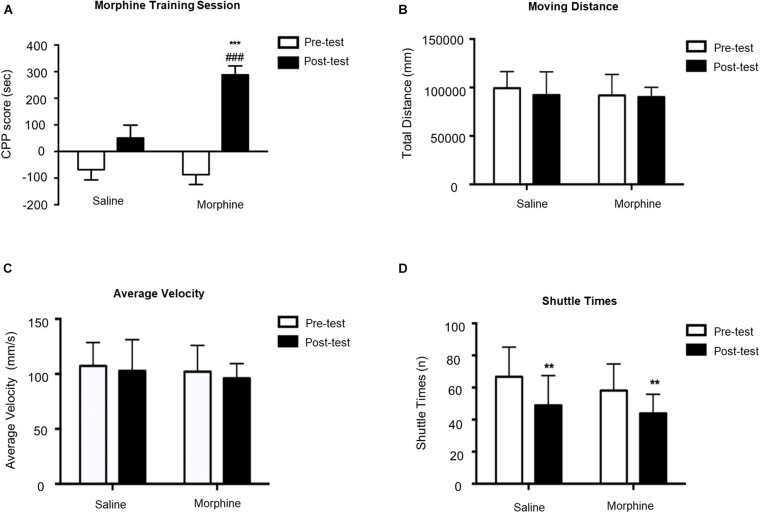
Morphine-induced conditioned place preference (CPP) acquisition on Day 10. **(A)** CPP score of pretest (Day 1) and posttest (Day 10). **(B)** Moving distance of pretest and posttest. **(C)** Average velocity of pretest and posttest. **(D)** Shuttle times of pretest and posttest. **p < 0.01 and ***p < 0.001 compared to the pretest; ^###^*p* < 0.001 compared to the posttest.

As shown in Figure 2, two-way ANOVA revealed significant effects of interaction [*F*_(1,84)_ = 10.66, *p* = 0.0016], time [*F*_(1,84)_ = 7.805, *p* = 0.0065], and group [*F*_(1,84)_ = 39.78, *p* < 0.0001]. Mice administered 10 mg/kg morphine had significantly higher CPP scores compared to saline-treated mice during posttest. We also measured the moving distance, average velocity, and shuttle times. We did not observe any difference in moving distance and average velocity, suggesting that morphine-induced memory did not influence explorative behavior of mice in the test apparatus. Two-way ANOVA on shuttle times indicated significant effect of time [*F*_(1,84)_ = 20.11, *p* < 0.0001] but no significant effect of interaction [*F*_(1,84)_ = 0.2481, *p* = 0.6197] or morphine [*F*_(1,84)_ = 3.615, *p* = 0.0607]. *T*-test showed that shuttle times in the posttest were dramatically decreased in both saline–treated (*t*_43_ = 3.219, *p* = 0.0024) and morphine-treated (*t*_41_ = 3.193, *p* = 0.0027) mice, suggesting their familiarity to the apparatus.

#### Extinction of Conditioned Place Preference

**FIGURE 3:**
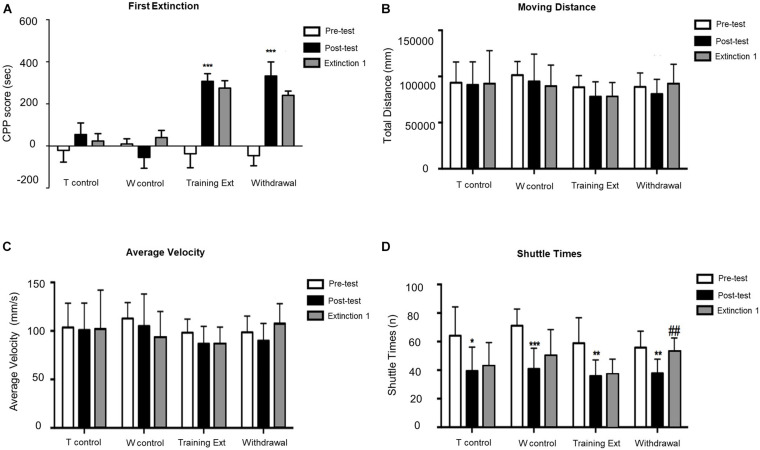
Morphine-induced conditioned place preference (CPP) was not extinguished after the first extinction session (Extinction 1). **(A)** CPP score of pretest (Day 1), posttest (Day 10), and extinction 1 test (Day 15). **(B)** Moving distance of pretest (Day 1), posttest (Day 10), and extinction 1 test (Day 15). **(C)** Average velocity of pretest (Day 1), posttest (Day 10), and extinction 1 test (Day 15). **(D)** Shuttle times of pretest (Day 1), posttest (Day 10), and extinction 1 test (Day 15). The Training Control group (*T control*) went through the same procedures as the Training Extinction group (*Training Ext*) except that the *T control* only received saline without morphine during conditioning; the Withdrawal Control group (*W control*) went through the same procedures as the *Withdrawal* group except that the *W control* only received saline without morphine during conditioning. *p < 0.05 compared to the pretest; **p < 0.01 compared to the pretest; ***p < 0.001 compared to the pretest; ^##^*p* < 0.01 compared to the posttest. *n* = 6–12 mice/group.

As shown in Figure 3A, mice with morphine-induced CPP went through the first extinction session with two different methods (training extinction vs. withdrawal extinction). No significant difference was observed between posttest and Extinction 1 in either training extinction groups (*p* > 0.05) or Withdrawal groups (*p* > 0.05), implying that the CPP still existed after the first extinction session. Additionally, the locomotion parameters were also monitored in all groups. As illustrated in Figures 3B,C, there were no significant differences in moving distance and average velocity, which revealed that the morphine-induced CPP and training extinction procedures did not affect the explorative behavior and general activity. Similar to CPP acquisition phase, two-way ANOVA on shuttle times, as shown in Figure 3D, revealed significant difference of time [*F*_(2,84)_ = 17.46, *p* < 0.0001] but non-significant effect of interaction [*F*_(6,84)_ = 1.306, *p* = 0.2633] or group [*F*_(3,84)_ = 1.305, *p* = 0.2748]. In the posttest, the decrease of shuttle times in the four groups revealed that the mice were more familiar with the test apparatus. In the first extinction session, the shuttle times in the training extinction group were basically unchanged, while those in the withdrawal group increased significantly with the constant total moving distance, indicating that morphine-induced memory was toward fading. Although the shuttle times may reveal a drug-related phenotype to some extent, it was not readout of drug-related memory. Therefore, there was a modest trending decrease of the CPP score during Extinction 1 in both groups.

**FIGURE 4:**
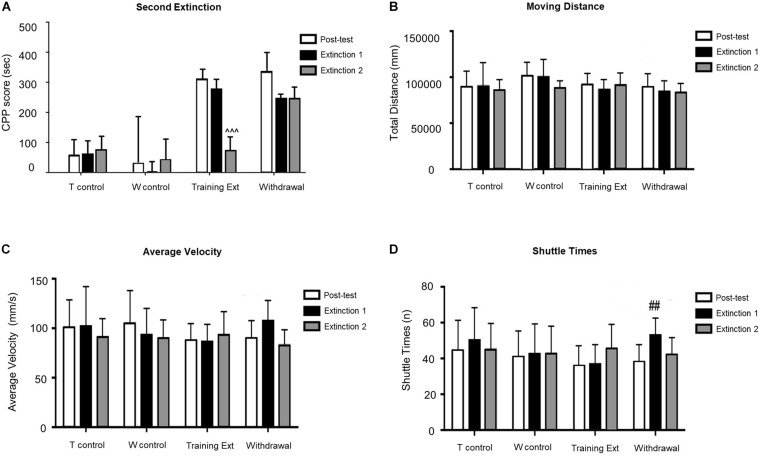
Morphine-induced conditioned place preference (CPP) was extinguished after the second extinction session (Extinction 2) in the extinction training group, but not in the withdrawal group. **(A)** CPP score of posttest (Day 10), extinction 1 test (Day 15), and extinction 2 test (Day 20). **(B)** Moving distance of each test. **(C)** Average velocity of each test. **(D)** Shuttle times of each test. The Training Control group (*T control*) went through the same procedures as the Training Extinction group (*Training Ext*) except that the *T control* only received saline without morphine during conditioning; the Withdrawal Control group (*W control*) went through the same procedures as the *Withdrawal* group except that the *W control* only received saline without morphine during conditioning. ^^^*p* < 0.001 compared to the posttest; ^##^*p* < 0.01 compared to the posttest. *n* = 6–12 mice/group.

As illustrated in Figure 4, mice with morphine-induced CPP went through the second extinction session in two different methods. CPP was extinguished in the training extinction group after two sessions of extinction, as *post hoc* test revealed a significant difference in CPP score between the second extinction and the posttest (*p* < 0.001). In contrast, withdrawal group showed no significant differences compared to the first extinction, implying that the CPP still existed by the end of the second extinction session (*p* > 0.05). In addition, the locomotion parameters did not change significantly in different phases of morphine-induced CPP of each group.

### Western Blotting Analysis

We evaluated the p-β-actin/β-actin ratio, Arc expression level, and p-ERK/ERK ratio in different brain regions during the different phases of morphine CPP. We also compared protein expression after two different methods of extinction test. Since all the control groups in the three different experimental groups were essentially the same, we used the control of morphine-induced CPP group as a representative to simplify the data analysis.

#### Alterations of p-β-actin/β-actin Ratio, Arc Expression, and p-ERK/ERK Ratio in the Nucleus Accumbens at Different Phases of Morphine-Induced Conditioned Place Preference

**FIGURE 5:**
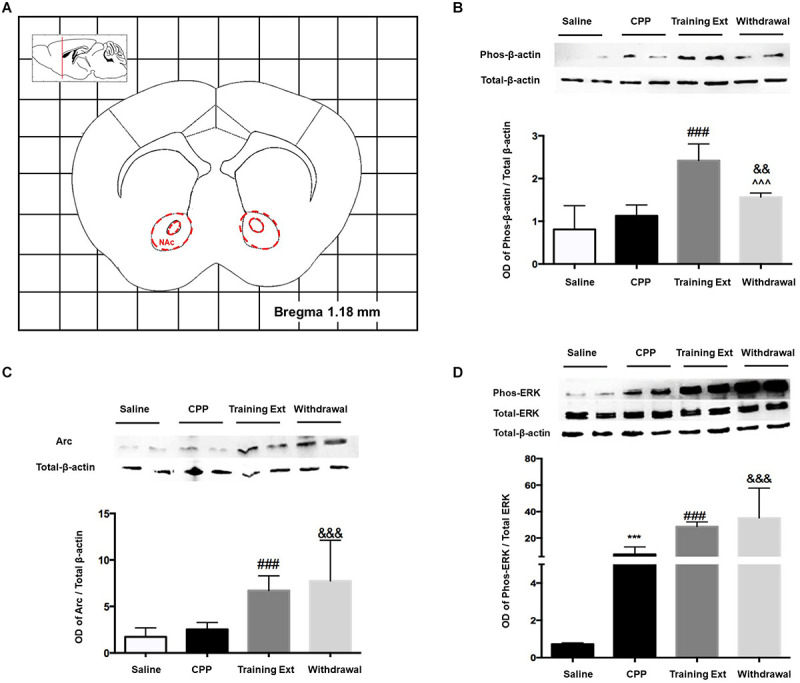
Alterations of the p-β-actin/β-actin ratio, Arc expression level, and p-ERK/ERK ratio in the nucleus accumbens (NAc) at different phases of the morphine-induced conditioned place preference (CPP). **(A)** Red letters and red dotted line in the coronal section inset indicate the dissection of samples from the NAc. Effects of different morphine-induced CPP phases on the **(B)** p-β-actin/β-actin ratio, **(C)** Arc expression level, and **(D)** p-ERK/ERK ratio in the NAc. The animals in the *Saline* or *CPP* group were sacrificed on posttest Day 10, while those in the Training Extinction group (*Training Ext*) and *Withdrawal* groups were sacrificed on Day 20. The *Training Ext* went through CPP procedures followed by saline injections in the training extinction; the *Withdrawal* group went through the CPP procedures followed by spontaneous extinction without any injections. ***p < 0.001 compared to the saline group; ^###^*p* < 0.001 compared to CPP acquisition group; ^^^*p* < 0.001 compared to training extinction group; ^&&^*p* < 0.01 and ^&&&^*p* < 0.001 compared to CPP acquisition group. *n* = 6–8 mice/group.

Activation of striatal neurons critically contributes to drug-associated memories, so firstly, we examined the protein expression in NAc. The red dotted line shown in Figure 5A indicated the dissection of samples from the NAc. As shown in Figure 5B, one-way ANOVA followed by Bonferroni test showed that the increase of p-β-actin/β-actin ratio [*F*_(3,20)_ = 22.05, *p* < 0.0001] in the training extinction group (*p* < 0.001) was more considerable than that of the withdrawal group (*p* < 0.01) compared to the CPP group. As illustrated in Figure 5C, the expression of Arc level [*F*_(3,20)_ = 9.270, *p* = 0.0005] did not change significantly in morphine-induced CPP group (*p* > 0.05) compared to the saline control, while it was remarkably upregulated in both the training extinction group and withdrawal group (*p* < 0.001) compared to the CPP group. Notably, the Arc alteration level tended to be consistent between the training extinction group and withdrawal group. As shown in Figure 5D, compared to saline control (*p* < 0.001), the p-ERK/ERK ratio dramatically increased after morphine exposure [*F*_(3,20)_ = 11.76, *p* = 0.0001]. Furthermore, after extinction training, the p-ERK/ERK ratio was further upregulated (*p* < 0.001). Taken together, our data indicated that morphine strongly regulated specific protein in the NAc.

#### Alterations of p-β-actin/β-actin Ratio, Arc Expression Level, and p-ERK/ERK Ratio in the Dorsal Hippocampus at Different Phases of Morphine-Induced Conditioned Place Preference

**FIGURE 6:**
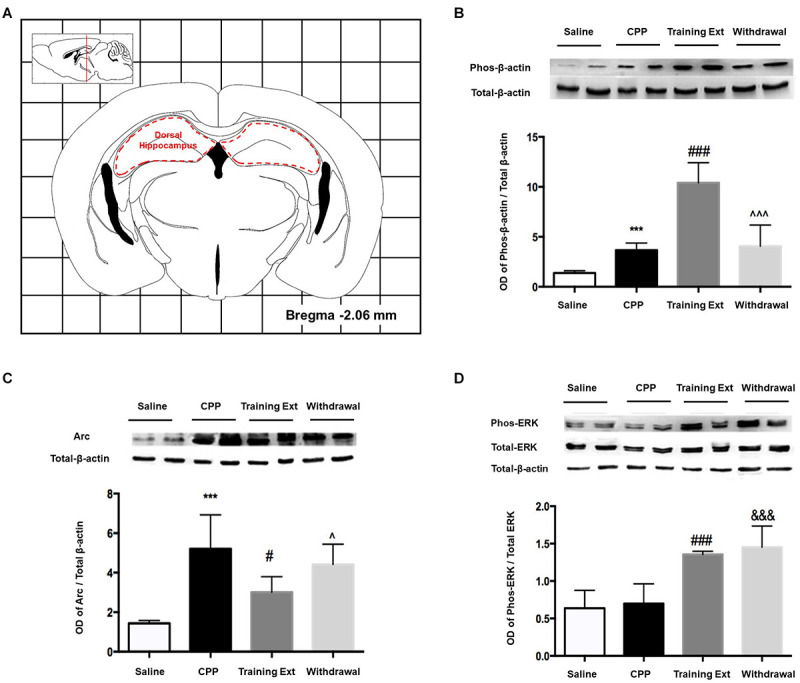
Alterations of the p-β-actin/β-actin ratio, Arc expression level, and p-ERK/ERK ratio in the dorsal hippocampus at different phases of the morphine-induced conditioned place preference (CPP). **(A)** Red letters and red dotted line in the coronal section inset indicate the dissection of samples from the dorsal hippocampus. Effects of different morphine-induced CPP phases on the **(B)** p-β-actin/β-actin ratio, **(C)** Arc expression level, and **(D)** p-ERK/ERK ratio in the dorsal hippocampus. The animals in the *Saline* or *CPP* group were sacrificed on posttest Day 10, while those in the Training Extinction group (*Training Ext*) and *Withdrawal* groups were sacrificed on Day 20. The *Training Ext* went through CPP procedures followed by saline injections in the training extinction; the *Withdrawal* group went through CPP procedures followed by spontaneous extinction without any injections. ***p < 0.001 compared to the saline group; ^#^*p* < 0.05 and ^###^*p* < 0.001 compared to CPP acquisition group; ^*p* < 0.05 and ^^^*p* < 0.001 compared to training extinction group; ^&&&^*p* < 0.001 compared to CPP acquisition group. *n* = 6–8 mice/group.

The red dotted line shown in Figure 6A indicated the dorsal hippocampus region, where we extracted bilateral brain tissue for experiments. As illustrated in Figure 6B, one-way ANOVA [*F*_(3,20)_ = 39.32, *p* < 0.0001] followed by Bonferroni test showed that p-β-actin/β-actin ratio in the morphine-induced CPP group was remarkably increased (*p* < 0.001) compared to its control group. The increase in training extinction was also significant (*p* < 0.001) compared to the CPP group, while in the withdrawal group, the ratio showed no significant change (*p* > 0.05). One-way ANOVA comparing protein levels of Arc showed a significant effect [*F*_(3,20)_ = 14.28, *p* < 0.0001]. Bonferroni test revealed that the Arc protein in the CPP group showed a noteworthy increase (*p* < 0.001) than that in the control group. After two training extinction sessions, the protein level showed a remarkable decrease (*p* < 0.05) compared to the CPP phase. Besides, the protein level in the withdrawal group was significant higher (*p* < 0.05) than that in the training extinction group, as shown in Figure 6C. One-way ANOVA comparing protein levels of p-ERK/ERK ratio demonstrated a significant effect [*F*_(3,20)_ = 43.78, *p* < 0.0001], as shown in Figure 6D. The increase in both extinction groups showed a significant difference compared to that of the CPP group (*p* < 0.001).

#### Alterations of p-β-actin/β-actin Ratio, Arc Expression Level, and p-ERK/ERK Ratio in the Medial Prefrontal Cortex at Different Phases of Morphine-Induced Conditioned Place Preference

**FIGURE 7:**
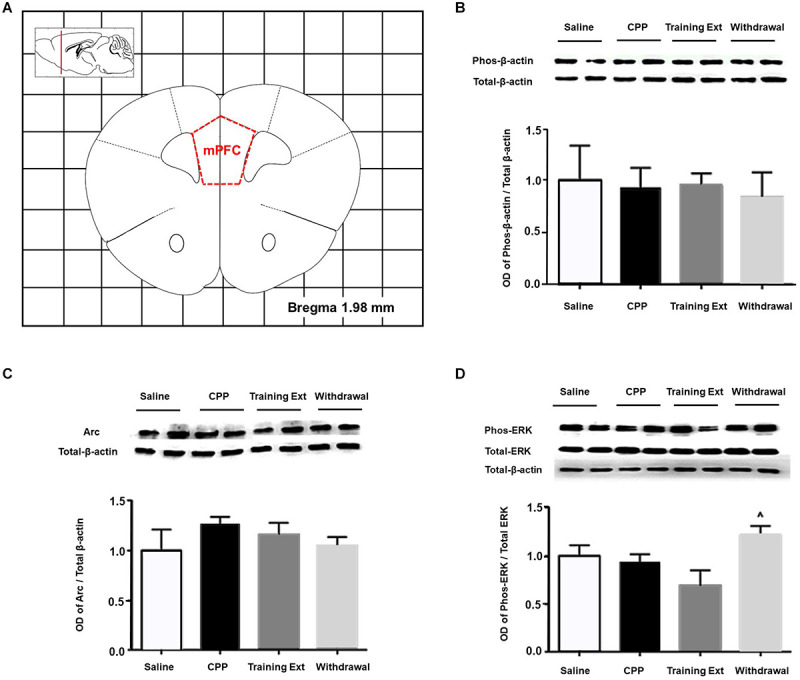
Alterations of the p-βactin/β-actin ratio, Arc expression level, and p-ERK/ERK ratio in the medial prefrontal cortex (mPFC) at different phases of the morphine-induced conditioned place preference (CPP). **(A)** Red letters and red dotted line in the coronal section inset indicate the dissection of samples from the mPFC. Effects of different morphine-induced CPP phases on the **(B)** p-β-actin/β-actin ratio, **(C)** Arc expression level, and **(D)** p-ERK/ERK ratio in the mPFC. The animals in the *Saline* or *CPP* group were sacrificed on posttest Day 10, while those in the Training Extinction group (*Training Ext*) and *Withdrawal* groups were sacrificed on Day 20. The *Training Ext* went through CPP procedures followed by saline injections in the training extinction; the *Withdrawal* group went through CPP procedures followed by spontaneous extinction without any injections. ^*p* < 0.05 compared to *Training Ext*. *n* = 6–8 mice/group.

The red dotted line shown in Figure 7A indicated the dissection of samples from the mPFC. As demonstrated in Figure 7B, one-way ANOVA [*F*_(3,20)_ = 45.32, *p* > 0.05] followed by Bonferroni test showed that p-β-actin/β-actin ratio in four different groups had no significant effect in the mPFC, as well as Arc expression level [*F*_(3,20)_ = 39.89, *p* > 0.05] shown in Figure 7C. As for p-ERK/ERK ratio [*F*_(3,20)_ = 16.15, *p* > 0.05], only the withdrawal group showed a significant increase (*p* < 0.05) compared to the training extinction group, as shown in Figure 7D.

#### Alterations of p-β-actin/β-actin Ratio, Arc Expression Level, and p-ERK/ERK Ratio in the Caudate Putamen at Different Phases of Morphine-Induced Conditioned Place Preference

**FIGURE 8:**
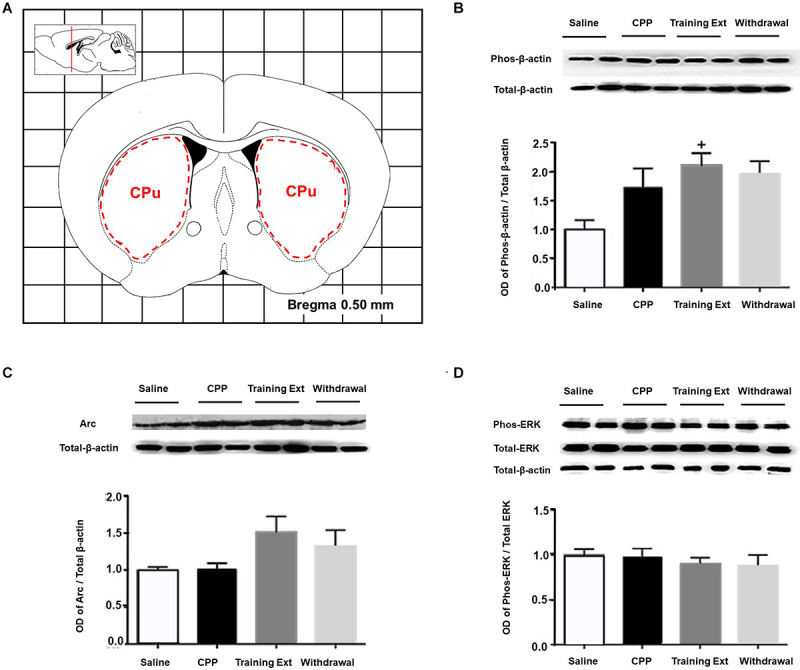
Alterations of the p-βactin/β-actin ratio, Arc expression level, and p-ERK/ERK ratio in the caudate putamen (CPu) at different phases of the morphine-induced conditioned place preference (CPP). **(A)** Red letters and red dotted line in the coronal section inset indicate the dissection of samples from the CPu. Effects of different morphine-induced CPP phases on the **(B)** p-β-actin/β-actin ratio, **(C)** Arc expression level, and **(D)** p-ERK/ERK ratio in the CPu. The animals in the *Saline* or *CPP* group were sacrificed on posttest Day 10, while those in the Training Extinction group (*Training Ext*) and *Withdrawal* groups were sacrificed on Day 20. The *Training Ext* went through CPP procedures followed by saline injections in the training extinction; the *Withdrawal* group went through CPP procedures followed by spontaneous extinction without any injections. ^+^*p* < 0.05 compared to the saline group. *n* = 6–8 mice/group.

Lastly, we measured the protein expression in the CPu. Figure 8A indicated the range of brain tissue extracted. One-way ANOVA [*F*_(3,20)_ = 22.16, *p* < 0.05] followed by Bonferroni test indicated that the increase of p-β-actin/β-actin ratio in the training extinction showed a dramatic increase compared to the saline group in the CPu (*p* < 0.05), as shown in Figure 8B. As for Arc expression level [*F*_(3,20)_ = 11.59, *p* > 0.05] and p-ERK/ERK ratio [*F*_(3,20)_ = 3.79, *p* > 0.05], no significant difference was observed in the four groups, as shown in Figures 8C,D.

## Discussion

The striatum, which was commonly divided into the dorsal part (CPu) and ventral part (NAc) based on anatomical localization, receives a prominent source of glutamatergic innervation from mPFC and hippocampus (Gonzales and Smith, 2015). Mounting evidence has demonstrated that these brain regions have been implicated in drug-associated memory (Arias-Carrion et al., 2010; Torregrossa et al., 2011), and many agents in these regions elicit aberrant drug-associated memory.

### Alteration in the Corticostriatal System

Most of the neurons within the striatum are medium spiny neurons. As previous studies have shown, drugs induced morphological changes of dendritic spines after withdrawal from chronic administration by means of modulating the actin cycle dynamic in the NAc (Zhao et al., 2019; Iino et al., 2020). Specifically, the underlying mechanisms are as follows. After withdrawal from repeated cocaine exposure, there was a reduction in the LIM kinase, which could inactivate cofilin, an actin-binding protein that controls the disassembly of actin filaments; alternatively, after chronic cocaine administration, cofilin is released from the inhibitory control of LIM kinase, potentiating the disassembly of actin filaments in individual monomers (Toda et al., 2006). Both pathways could result in an increasing amount of G-actin, specifically, β-actin.

There is, however, a paucity of studies directly investigating the role of β-actin in opiate extinction. Previous study examined dendritic morphology after extinction training in an opiate CPP model and demonstrated a reduction of dendritic complexity (Leite-Morris et al., 2014). These results may seem at odds with the present findings, and the discrepancy may be attributed to two reasons. It is known that cocaine and morphine induce changes in dendritic spine morphology *via* two phases. The first phase is characterized by an initial increase in spine head diameter and increases in AMPA receptor expression, followed by a second stage of spine head diameter retraction and reduction of the AMPA receptor expression in spines (Quintero, 2013). Our extinction training went through just two trials, almost 10 days from posttest to tissue sampling, while Kimberly A performed three trials, 16 days in total before morphological detection. Thus, the contrasting findings just reflect dendritic spine morphology and actin dynamics at different time points. On the other hand, the reduction in dendritic complexity, characterized by a decreased number of dendritic intersections, shorter total dendritic length, as well as decreased c-Fos protein expression, appears to result primarily from depolymerization of F-actin into monomeric forms, resulting in a transient rise of β-actin, which was observed in our experiment. Hence, we postulated that the relationship between β-actin increase and F-actin formation might not be a simple linear positive correlation.

Previous study has demonstrated that Arc/Arg3.1 protein expression in the NAc core is critical for the acquisition, expression, and reinstatement of morphine CPP (Lv et al., 2011), as well as its role in the NAc shell during the reconsolidation (Lv et al., 2015). However, little is known about the function of this protein in the extinction period of drug-induced long-term memory. The results from our experiments revealed that, when re-exposed to the context after 8 days of extinction training, the significant place preference for the morphine-paired chamber disappeared in the training extinction group but not in the withdrawal group, both of which were accompanied by an increase in Arc/Arg3.1 protein expression in the NAc. The elevation of Arc/Arg3.1 protein in both groups was in accordance with augmentation of β-actin. Based on these findings, we hypothesized that there might be a positive correlation between Arc/Arg3.1 protein expression and β-actin in the NAc. It is noteworthy that there is no significant increase in the expression of Arc/Arg3.1 after CPP acquisition, while a previous study revealed that reactivation of morphine context memory after the expression test dramatically enhanced the Arc/Arg3.1 protein level, accompanied by increases in p-ERK1/2, specifically in the NAc shell (Lv et al., 2011). This discrepancy is most likely due to the different harvest time points, i.e., immediately after CPP test in the current study vs. 2 h after the expression test in the previous study. The Arc gene belongs to immediate early gene (IEG) group (Fujiki et al., 2020). Similar to other IEGs, such as c-fos, the expression of Arc is temporally specific, as it demonstrated that a single dose of morphine increased Arc/Arg3.1 protein levels in the NAc core 2 h after morphine administration, rather than 1, 3, and 4 h (Lv et al., 2011). The 2-h interval is just for Arc mRNA to selectively target, translate, and be enriched at recently activated synaptic sites (Liu et al., 2012). Translation of the dendritically localized Arc mRNA is required for consolidation of long-term potentiation (LTP) and stabilization of nascent polymerized actin, which are believed to underlie synaptic plasticity in long-term memory (Bramham, 2008).

Abuse of drugs, including morphine and cocaine, increased ERK phosphorylation in the NAc (Valjent et al., 2004; Xu et al., 2012). Consistent with these reports, our results showed that an upregulation of p-ERK1/2 was observed during the establishment phase and extinction phase, including training extinction group and withdrawal group. Slightly different from the researches mentioned above, Yuan Ma et al. (2014) reported that morphine decreased the level of ERK1/2 mRNA during the establishment phase and increased during the extinction phase. Considering the structural and functional complexity of NAc, as well as testing approaches as Western blotting vs. RT-PCR, it is hard to reach a consensus on the overall effect of morphine in the NAc during CPP. Besides, the local activation of the ERK–CREB signal pathway, as an upstream mechanism of Arc/Arg3.1, is required for Arc/Arg3.1 in the NAc shell, mediating morphine-associated context memory (Lv et al., 2015).

In conclusion, β-actin in the NAc mediates morphine-associated context memory *via* upregulating the level of Arc/Arg3.1. In this process, the local activation of the ERK signal pathway, as an upstream mechanism of Arc/Arg3.1, is required.

### Alteration in the Dorsal Hippocampus

Hippocampus activity is required for both learning to associate specific contexts with reinforcing availability and spatial learning (Li et al., 2020). Stabilization of LTP depends on multiple signaling cascades linked to actin polymerization (Fedulov et al., 2007). Hippocampal LTP is accompanied by enhanced F-actin content within the dendritic spine, which is essential for late LTP maintenance in unanesthetized freely moving rats (Fukazawa et al., 2003). Besides, actin assembly plays an essential role in the molecular process underlying hippocampal LTP. Moreover, actin turnover in dendritic spines influences spine development, morphology, and plasticity, with functional consequences on learning and memory formation.

The above findings suggested that β-actin, with its modulator, Arc/Arg3.1, might initiate changes in synaptic anatomy while maintaining LTP. An upregulation of β-actin was observed in both expression and extinction phases compared with saline group. As we all know, both development and extinction of CPP reflected formation of new memory, while the withdrawal group reflected that new memory had not been well established. In line with our behavioral data, β-actin was elevated in two relevant groups and decreased in the withdrawal group. Since β-actin is sensitive to alterations in hippocampus, it may be regarded as a marker of neuronal activation in this brain region.

Previous studies have demonstrated that Arc controls LTP consolidation *via* regulating of local actin polymerization in the dentate gyrus *in vivo* and amygdala modulates inhibitory avoidance and contextual fear memories by regulating the expression of Arc gene and/or protein in the hippocampus (Hou et al., 2009). However, elevation of Arc was detected by neither immunohistochemistry nor Western blotting in the dorsal hippocampus, which suggested that the amygdala modulated aversive memory of morphine withdrawal *via* actin rearrangements but not Arc protein expression in the dorsal hippocampus (Hou et al., 2009). In parallel with these findings, our results revealed a lack of upregulation of Arc in the extinction phase despite a significant elevation in CPP expression phase, which is distinct from modulation of memories by the NAc.

It is also noticeable that there are challenging studies dealing with ERK activity within the hippocampus. In a previous study, Yuan Ma et al. (2014) showed that ERK1 and ERK2 mRNA levels were downregulated in the hippocampus during the three phases of CPP. Earlier study showed that chronic methamphetamine (METH) treatment did not alter ERK phosphorylation in mouse hippocampus (Su et al., 2013). Furthermore, another investigation indicated that there was no significant alteration of p-ERK in the hippocampus and PFC after CPP induced by chemical stimulation of the lateral hypothalamus (Haghparast et al., 2011). Concurrent with recent investigations, our results showed that morphine did not change the level of activated ERK1/2 during the establishment phase, but abruptly increased it in the extinction phase. Thus, it could be taken into consideration that ERK in the dorsal hippocampus is a crucial player for CPP extinction, but not for CPP development.

It also should be noted that actin rearrangements and its modulator Arc in the NAc were delayed than that in the hippocampus, as no fluctuations of their expression were observed during the CPP development period. This may suggest that the hippocampus may impose some regulation on NAc anatomically and functionally, which requires further investigation.

In summary, our work illustrated that morphine altered β-actin and Arc, ERK1/2 expression in brain regions during both CPP establishment phase and extinction phase, leading to brain region-specific changes. Those proteins were in a different level related to different behavioral stages in the NAc and dorsal hippocampus, respectively. Phosphorylation of β-actin and Arc contribute to memory formation, while p-ERK was affected by drug reward response from morphine and may affect the response in return. These findings contribute to a deeper understanding of the potential mechanisms of cytoskeletal protein in cellular and molecular processes underlying drug-seeking behaviors. Last but not least, F-actin and G-actin should also be detected in future studies to reflect actin rearrangement more directly. It is expected that the actin dynamics in the process of drug-induced behavior will provide more insights into the treatment of drug addiction.

## Data Availability Statement

The original contributions presented in the study are included in the article/supplementary material, further inquiries can be directed to the corresponding author/s.

## Ethics Statement

The animal study was reviewed and approved by Xi’an Jiaotong University Laboratory Animal Administration Committee.

## Author Contributions

XY, YZ, and CY designed the research. XY, YW, and YZ performed the experiments. FG, JY, and ZY provided the technical support. XY and YW analyzed the data. XY and YZ wrote the manuscript. CY edited the manuscript. All the authors approved the manuscript for publication.

## Conflict of Interest

The authors declare that the research was conducted in the absence of any commercial or financial relationships that could be construed as a potential conflict of interest.
